# Kinematic versus mechanical alignment for primary total knee arthroplasty with minimum 2 years follow-up: a systematic review

**DOI:** 10.1051/sicotj/2020014

**Published:** 2020-06-17

**Authors:** Elliot Sappey-Marinier, Adrien Pauvert, Cécile Batailler, John Swan, Laurence Cheze, Elvire Servien, Sébastien Lustig

**Affiliations:** 1 FIFA medical center of excellence, Orthopaedics Surgery and Sports Medicine Department, Croix-Rousse Hospital, Hospices Civils de Lyon 103 Grande rue de la Croix Rousse 69004 Lyon France; 2 Univ Lyon, Claude Bernard Lyon 1 University, IFSTTAR, LBMC UMR_T9406 69622 Lyon France; 3 LIBM – EA 7424, Interuniversity Laboratory of Biology of Mobility, Claude Bernard Lyon 1 University 69100 Villeurbanne France

**Keywords:** Total knee arthroplasty, Total knee replacement, Mechanical alignment, Kinematic alignment, Systematic review

## Abstract

*Purpose*: The aim of this study was to perform a systematic review of the literature to determine whether there are any clinical or radiological differences in mechanically aligned Total Knee Arthroplasty (TKA) compared with kinematically aligned TKA. *Methods*: This study included retrospective cohort studies, prospective randomized controlled trials (PRCTs) and prospective cohort studies comparing clinical and radiological outcomes, and complications in TKA with kinematic alignment (KA) and mechanical alignment (MA). All studies had a minimum follow-up of 2 years. *Results*: Five PRCTs published between 2014 and 2020 were included. These studies showed a low risk of bias and were of very high quality. We did not find a superiority of KA compared to MA technique for clinical and radiological outcomes, except in one study which showed a significant difference favoring KA between the two groups for all clinical scores. *Conclusion*: We found that KA in TKA achieved clinical and radiological results similar to those of MA. The complication rate was not increased for KA TKAs. Studies with longer follow-up and larger cohorts are required to prove any benefit of KA technique over MA technique.

## Introduction

Total knee arthroplasty (TKA) is an effective method for the treatment of severe osteoarthritis of the knee [1]. One of the foundations of a successful TKA is the restoration of neutral knee alignment [2]. Mechanical alignment (MA) in TKA aims to position both femoral and tibial components perpendicular to the mechanical axis of each bone. This allows to obtain a hip-knee-ankle (HKA) angle of the limb of 180° considered as neutral under static weightbearing conditions [3]. This is a fundamental principle of TKA, willing to obtain a symmetric balanced load distribution between the medial and lateral compartments in order to minimize wear and potential component loosening [4–8].

However, this situation differs from the native knee. Indeed, the tibial coronal alignment averages 3° varus and the mean femoral coronal alignment is of 3° valgus relative to the mechanical axis. Moreover, there is a wide individual variation in limb alignment. Bellemans et al. showed that more than 30% of male non-arthritic patients had a constitutional varus angle of > 3° [9]. Also, Hirschmann et al. showed a wide distribution of femoral and tibial coronal alignment in young non-osteoarthritic knees [10]. If such patients undergo TKA according to MA principles, medial or lateral soft tissue adjustments may be required [9, 11, 12], which could explain the results of various studies [13, 14] showing that up to 20% of patients are dissatisfied after TKA.

Therefore, kinematic alignment (KA) for TKA, in the wider sense, has been proposed as an alternative approach to neutral mechanical alignment [15]. This alternative alignment approach is more patient-specific and is defined by four alignment strategies: anatomic alignment, adjusted mechanical alignment, kinematic alignment, and restricted alignment techniques [16]. In different studies assessing this alternative alignment, there still persists some ambiguities on which of the four techniques was analyzed, while all authors name it kinematic alignment in the wider sense. KA aims to position TKA implants to match the native anatomy of each patient. It produces anatomic rather than systematic component positions, more physiological joint line obliquity and more physiological knee kinematics [17]. Defenders of KA suggest that this will improve clinical results in terms of pain and function compared to MA technique by reducing the need for ligament releases and improve soft tissue balancing [18–20]. However, there are concerns on implant survival with implants positioned in a different way from traditional targets [5, 21–23]. Several systematic reviews were performed comparing KA and MA techniques at an early follow-up [2, 24–29]. No review has been performed comparing KA and MA with a minimum follow-up of 2 years. Thus, the aim of this study was to perform a systematic review of the literature to determine whether there are any clinical or radiological differences in mechanically aligned TKA compared with kinematically aligned TKA. Our outcome variables included clinical rating scores, radiological outcomes, survivorship, complications or gait analysis at a minimum follow-up of 2 years.

## Methods

### Literature search strategy

For this study, the Preferred Reporting Items for Systematic Reviews and Meta-Analyses (PRISMA) guidelines were followed [30]. A first electronic search was performed using PubMed, Ovid Medline and Cochrane library from their dates of inception to the 2nd March 2020. To maximize sensitivity of the search strategy the authors combined the terms “knee”, “arthroplasty”, “replacement”, “kinematic”, “kinematically”, “mechanical”, “mechanically”, and “alignment” when searching in the title, abstract, keywords, and MeSH fields. A secondary search was performed examining the references cited in the articles found in the primary search. All articles were reviewed by two authors independently following this systematic approach. Each reviewer was blinded with regard to the determination of the other reviewer. Ethical approval was not necessary in this study as it only analyzed current studies and did not collect individual patient data. No external funding was received for this project.

### Selection criteria

This study included retrospective cohort studies, prospective randomized controlled trials (PRCTs), and prospective cohort studies comparing clinical and radiological outcomes with KA and MA in TKA. For inclusion, studies contained a KA group and a MA group with a minimum of 10 TKAs in each group and a minimum follow-up of 2 years. Final analysis involved studies reporting clinical outcome scores, survivorship, complications, or postoperative radiographic alignment between kinematic and mechanical alignment prostheses. When several studies reported the results of the same patient series with different follow-ups, only the last study with the longest follow-up was analyzed. All publications included were limited to those written in English language, involving human subjects, and full-text availability for the articles. Case reports, duplicate studies, letters, noncomparative studies, conference presentations, expert opinions, and reviews were excluded.

### Data extraction

All the relevant data were extracted from article text, figures, and tables. Two investigators independently reviewed and extracted data from the retrieved articles. Discrepancies at the full-text stage were resolved by consensus between the two reviewers. If a consensus could not be reached, a third, more senior reviewer helped to resolve the discrepancy. The two reviewers collected information regarding the authors, publication origin, publication date, patient demographics (age, gender, sample size, and body mass index (BMI)), surgical methods, prosthetic designs, and outcome measurements.

The primary outcomes were the clinical and radiological results. The clinical results included the Knee Society score (KSS) [31], Western Ontario and McMaster Universities Osteoarthritis Index (WOMAC) [32], Oxford Knee score (OKS) [33], range of motion (ROM), Hospital for special surgery (HSS) score, Forgotten Joint Score (FJS) [34], Knee Injury and Osteoarthritis Outcome Score (KOOS JR) [35], a visual analog score for satisfaction, and the University of California Los Angeles (UCLA) Activity Score [36].

### Quality assessment

A risk-of-bias evaluation was performed using the Cochrane Collaboration tool [37]. Seven domain-based evaluations related to risk of bias were performed, including evaluation for random sequence generation (selection bias), allocation concealment (selection bias), blinding of the participants and personnel (performance bias), blinding of the assessors (defection bias), incomplete outcome data (attrition bias), and selective reporting (reporting bias) and other biases. The overall quality of each study was evaluated as a “low risk of bias”, a “high risk of bias”, or an “unclear risk of bias”.

A modified Jadad score was used for the quality evaluation of PRCTS including data analysis, blinding, randomization, withdrawal, adverse reactions, and inclusion criteria. Low-quality studies scored from 0 to 3 and high-quality studies scored from 4 to 8.

### Statistical analysis

Descriptive statistics, such as means, ranges, and measures of variance (standard deviations, 95% confidence intervals (CI)), are presented where applicable. No meta-analysis was performed.

## Results

**Figure 1:**
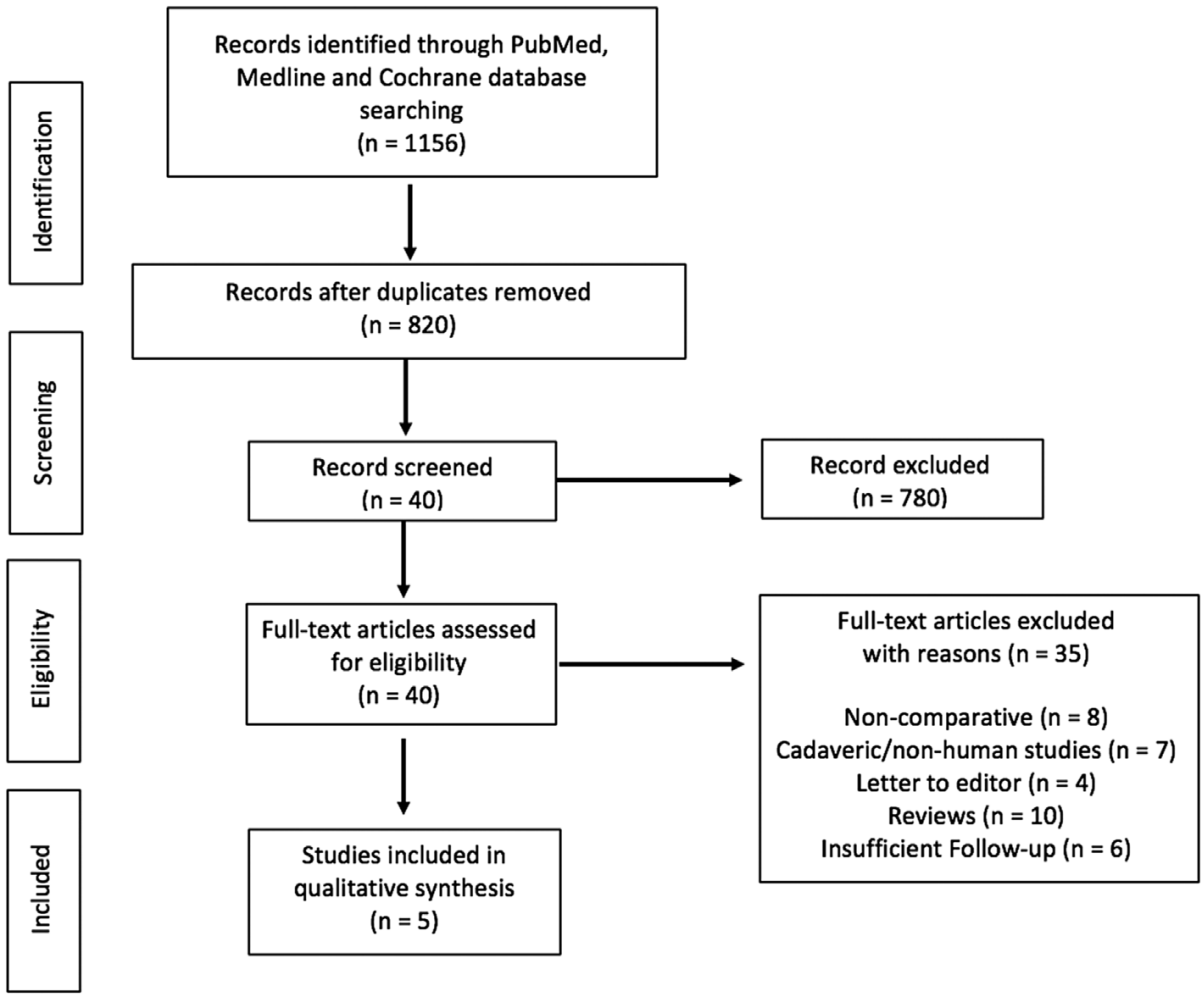
Flow chart.

The selection procedure is shown in Figure 1. A total of 1156 studies were identified by using our primary and secondary search strategy. After exclusion of duplicate studies, a total of 820 studies remained for further screening. Examination of title/abstracts excluded 780 records, and a further 35 were excluded. Thus, 5 PRCTs were included [18, 38–41] and were published between 2014 and 2020. Characteristics of the studies included are reported in Table 1.

**Table 1:** Characteristic of the studies.

Studies	Location	Study design	Minimum follow-up (m)	Sample size		Mean age		Female		BMI (mean)		Prosthesis design	Operative method	Clinic al measurements	Radiological measurements	Other measurements
				KA	MA	KA	MA	KA	MA	KA	MA					
Dossett et al. [18]	United States	PRCT	24	44	44	66	66	41	38	29	32	KA: Vanguard, CR, FB, SR, cemented, all PR	KA: PSI	OKS, WOMAC, KSS, ROM	HKA, AKA, JLOA, FMA, TMA	Complications
												MA: Vanguard, CR, FB, SR, cemented, all PR	MA: Conventional instruments			
Yeo et al. [39]	South Korea	PRCT	96	30	30	72	74	27	25	27	26	KA: NexGen, CR, FB, MR, cemented, partial PR	KA: ROBDOC system, robotic aassisted	HSS, WOMAC, KSS, ROM	HKA, FMA, TMA, TS	Gait analysis
												MA: NexGen, CR, FB, MR, cemented, partial PR	MA: Robotic aassisted			
Laende et al. [40]	Canada	PRCT	24	24	23	64	63	16	17	36	34	KA: Triathlon, CR, FB, SR, cemented, all PR	KA: PSI	OKS, satisfaction, UCLA	HKA, MPTA	Tibial migration
												MA: Triathlon, CR, FB, SR, cemented, all PR	MA: Computer navigation			
McEwen et al. [41]	Australia	PRCT	24	41	41		65	NA			31	KA: Triathlon, CR, FB, SR, cementless femur, cemented tibia, partial PR	KA: computer navigation	OKS, FJS, KOOS, JR, ROM	HKA, FMA, TMA, TS, JLOA, JLCA, PTA	Intraoperative gap laxity, soft tissue release, 4 specific clinical questions, complications
												MA: Triathlon, CR, FB, SR, cementless femur, cemented tibia, partial PR	MA: computer navigation			
Young et al. [38]	New Zealand	PRCT	60	47	48	72	70	25	26	30	31.5	KA: Triathlon, CR, FB, SR, cemented, partial PR	KA: PSI	OKS, WOMAC, FJS, KSS, VAS	HKA, FMA, TMA, TS	Intraoperative gap laxity, soft tissue release, complications
												MA: Triathlon, CR, FB, SR, cemented, partial PR	MA: Computer navigation			

NA: Not Applicable, PRCT: Prospective Randomized Controlled Trial, KA: Kinematic Alignment, MA: Mechanical Alignment, CR: Cruciate-Retaining, FB: Fixed-Bearing, SR: Single Radius, MR: Multi-Radius, PR: Patella Resurfacing, PSI: Patient-Specific Instrument, OKS: Oxford Knee Score, WOMAC: Western Ontario and McMaster Universities Osteoarthritis Index, KSS: Knee Society Score, ROM: Range of Motion, FJS: Forgotten Joint Score, VAS: Visual Analogue Scale, HSS: Hospital for Special Surgery knee score, UCLA: University of California at Los Angeles Loneliness score, KOOS JR: Knee Injury and Osteoarthritis Outcome Score Junior, HKA: Hip-Knee-Ankle angle, AKA: Anatomic Knee Angle, JLOA: Joint Line Orientation Angle, FMA: Femoral component relative to Mechanical Axis, TMA: Tibial component relative to Mechanical Axis, TS: Tibial Slope, MPTA: Medial Proximal Tibial Angle, JLCA: Joint Line Convergence Angle, PTA: Patellar Tilt Angle.

### Risk of bias and quality of evidence

**Figure 2:**
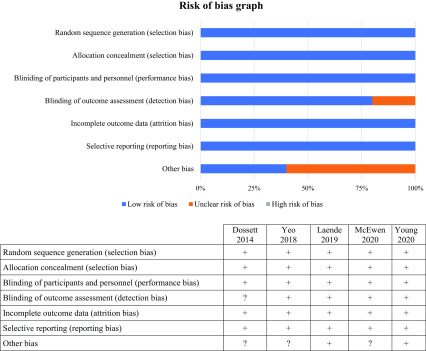
Risk of bias graph; “+ or plus” indicates a low risk of bias; “− or minus” indicates a high risk of bias; and “? or question mark” indicates unclear of unknown risk of bias.

Risk-of-bias assessment results of the studies are reported in Figure 2. There was an unclear risk of bias in one study in blinding the outcome assessment (detection bias). There was an unclear risk of bias for the category “other bias” in three studies. We did not find other bias in the included studies. The quality evaluation scores of the studies are shown in Table 2. The studies included in this systematic review were given scores of 8. Thus, after examination, the five included studies had a low risk of bias and were of very high quality.

**Table 2:** Modified-Jadad score.

	Dossett et al. [18]	Yeo et al. [ 39]	Laende et al. [40]	McEwen et al. [41]	Young et al. [38]
Was the study described as randomized?	Yes	Yes	Yes	Yes	Yes
Was the method of randomization appropriate?	Yes	Yes	Yes	Yes	Yes
Was the study described as blinded?	Yes	Yes	Yes	Yes	Yes
Was the method of blinding appropriate?	Yes	Yes	Yes	Yes	Yes
Was there a description of withdrawals or dropouts?	Yes	Yes	Yes	Yes	Yes
Was there a clear description of the inclusion/exclusion criteria?	Yes	Yes	Yes	Yes	Yes
Was the method used to assess adverse effects described?	Yes	Yes	Yes	Yes	Yes
Was the method of statistical analysis described?	Yes	Yes	Yes	Yes	Yes
Total score	8	8	8	8	8

### Clinical results

Four studies did not find any significant difference between the two groups (mechanical or kinematic alignment) for all the scores [38–41]. One study found a significant difference between the two groups for all the scores [18]. In this study, patients who underwent kinematically aligned TKA had significantly better scores for pain, function, and ROM than those who underwent mechanically aligned TKR. Patients in KA group had less pain according to the OKS and WOMAC scores. The mean flexion was increased by 8.5° in the KA group compared to the MA group. All clinical results are reported in Table 3.

**Table 3:** Clinical results at the last follow up (*SD* = standard deviation).

Studies	Clinical assessment	Kinematic alignment	Mechanical alignment	*p*-value
		Mean (SD) (range)	Mean (SD) (range)	
Dossett et al. [18]	OKS	40 (10.2) (15 to 48)	33 (11.1) (13 to 48)	0.005
	WOMAC	81 (20.3) (33 to 100)	70 (22.6) (23 to 100)	0.005
	KSS	160 (31.9) (93 to 200)	137 (37.9) 64 to 200)	0.005
	Flexion	121 (10.4) (100 to 150)	113 (12.5) (80 to 130)	0.002
Yeo et al. [39]	HSS	94.8 (5.5)	93.2 (8.0)	> 0.05
	WOMAC	79.6 (1.8)	80.7 (1.9)	> 0.05
	KSS	140.2 (16.6)	137.6 (16.1)	> 0.05
	Flexion	129 (11.5)	125 (11.5)	> 0.05
Laende et al. [40]	OKS	31 (7.8)	30 (8.6)	0.61
	Satisfaction	94 (12.9)	91 (19.0)	0.49
	UCLA	6.1 (1.9)	5.9 (2.0)	0.60
McEwen et al. [41]	OKS	44.4 (4.3) (30 to 48)	44.1 (4.1) (32 to 48)	0.58
	KOOS JR	89.6 (12.9) (55 to 100)	88.5 (13.7) (45 to 100)	0.69
	FJS	79.9 (23.5) (0 to 100)	79.6 (19.4) (19 to 100)	0.54
	Flexion	127 (10) (101 to 154)	127 (11) (105 to 150)	0.98
Young et al. [38]	OKS	41.4 (7.2)	41.7 (6.3)	0.99
	WOMAC	86.1 (15.5)	89.1 (15.3)	0.65
	FJS	68 (28.8)	74.4 (23.6)	0.29
	KSS	155.6 (30.6)	160.9 (25.8)	0.65
	VAS	78.2 (16.5)	78.4 (17.1)	0.99

OKS: Oxford Knee Score, WOMAC: Western Ontario and McMaster Universities Osteoarthritis Index, KSS: Knee Society Score, ROM: Range of Motion, FJS: Forgotten Joint Score, VAS: Visual Analogue Scale, HSS: Hospital for Special Surgery knee score, UCLA: University of California at Los Angeles Loneliness score, KOOS JR: Knee Injury and Osteoarthritis Outcome Score Junior.

### Radiological results

For all studies, postoperative HKA was not significantly different between both groups. However, in each study, the tibial component was placed significantly more in varus (from 2° to 3.5°) in the KA group compared to the MA group. Also, four studies reported the femoral component positioning and it was significantly more in valgus (from 1.3° to 1.8°) in the KA group compared to the MA group. All radiographic results are reported in Table 4.

**Table 4:** Radiological results at the last follow up (*SD* = standard deviation).

Studies	Radiological assessment	Kinematic alignment	Mechanical alignment	*p*-value
		Mean (*SD*) (range)	Mean (*SD*) (range)	
Dossett et al. [18]	HKA	−0.1 (2.8) (−8.5 to 7.7)	0.1 (2.5) (−4.9 to 8.9)	0.818
	AKA	3.5 (2.3) (0.1 to 9.5)	2.9 (2.3) (−1.2 to 8.0)	0.233
	JLOA	2.0 (2.0) (−3.1 to 6.6)	0.1 (2.7) (−4.1 to 8.4)	<0.001
	FMA	1.3 (2.0) (−2.4 to 6.5)	−0.8 (2.7) (−5.8 to 6.3)	<0.001
	TMA	−2.2 (2.6) (−8.7 to 4.4)	0.0 (2.1) (−6.4 to 3.8)	<0.001
Yeo et al. [39]	HKA	0.1 (2.0)	−0.3 (1.7)	n.s
	FMA	1.7 (1.9)	−0.5 (0.4)	0.04
	TMA	−2.5 (1.7)	0.1 (0.4)	0.03
	TS	−7.5 (2.8)	−6.4 (1.0)	n.s
Laende et al. [40]	HKA	−2.3 (2.6)	−2.1 (2.1)	0.75
	MPTA	−3.3 (2.0)	−0.8 (1.7)	<0.001
McEwen et al. [41]	HKA	−1.0 (2.4) (−6.0 to 3.9)	−0.2 (2.1) (−4.5 to 5)	0.097
	FMA	1.8 (2.0) (−2.5 to 5.5)	−0.1 (0.6) (−1.5 to 1)	<0.001
	TMA	−2.5 (1.3) (−5.5 to 0.5)	−0.3 (0.4) (−1 to 0.5)	<0.001
	TS	3.1 (1.8) (−0.5 to 7.5)	3.2 (1.8) (−0.5 to 7.5)	0.713
	JLOA	−0.9 (2.6) (−6.4 to 4.9)	0.8 (2.1) (−5.2 to 5.2)	0.002
	JLCA	0.7 (0.7) (0.0 to 3.3)	0.9 (0.6) (0 to 2.2)	0.141
	PTA	5.7 (5.8) (−2.9 to 20.1)	4.4 (4.9) (−6 to 14.8)	0.278
Young et al. [38]	HKA	−0.4 (3) (−11 to 6)	−0.7 (2) (−5 to 4)	0.6
	FMA	2 (2.5) (−4 to 6)	0.5 (1.6) (−3 to 4)	0.002
	TMA	−3 (3) (−10 to 4)	−0.7 (1.8) (−6 to 2)	<0.001
	TS	−4 (2.5) (−10 to 2)	−1.3 (2) (−7 to 3)	<0.001

HKA: Hip-Knee-Ankle angle, AKA: Anatomic Knee Angle, JLOA: Joint Line Orientation Angle, FMA: Femoral component relative to Mechanical Axis, TMA: Tibial component relative to Mechanical Axis, TS: Tibial Slope, MPTA: Medial Proximal Tibial Angle, JLCA: Joint Line Convergence Angle, PTA: Patellar Tilt Angle.

### Other measurements

Among studies, three reported postoperative complication rate between MA and KA groups at the last follow-up and no significant difference was found [18, 38, 41]. One study compared gait analysis, and no significant difference was found between the KA and MA groups [39]. Laende et al. [40] compared the tibial component migration at two years which was not significantly different between both groups. McEwen et al. [41] in their study found that significantly more patients preferred their KA joint and patients were visually insensitive to HKA angle asymmetry.

Two studies [38, 41] reported intraoperative gap laxity values and soft tissue releases. Both studies showed the same results with no significant difference being found for the gap laxity values between KA and MA groups. However, a significant increase in the number of releases was necessary to produce the target laxity values in the MA group compared to the KA group in both studies.

## Discussion

This review is the first to isolate all comparative studies between KA and MA groups with a minimum follow-up of 2 years. All studies included were level I and therefore of very high quality. The most important findings of this review were to show comparable or superior clinical outcomes of KA TKA to those of MA TKA. The limb and knee alignment in KA TKA were similar to those of MA TKA, still the femoral component was placed slightly more in valgus and the tibial component was implanted in mild varus in KA TKA. The joint line orientation angle (JLOA) of the KA TKA was quite parallel to the floor and closer to the native knee than the JLOA of the MA TKA. Moreover, the complication rate was not increased for KA TKA.

More individualized alignment strategies in TKA, such as kinematic alignment, appears to be one of the “hot” topics in TKA procedures [42]. This is particularly interesting to surgeons convinced by the fundamental principles of mechanically aligned TKA, and yet willing to improve clinical results after TKA. Indeed, according to previous studies [19, 43], good performance of knee functional recovery is directly associated with patient satisfaction and implant survival. KA has gained extensive interest according to high encouraging patient-reported results [17, 19]. McEwen et al. [41] showed that significantly more patients preferred their KA joint compared to their MA joint. The satisfactory results reached with KA TKA could be attributable to the attempt to replicate the alignment of the pre-osteoarthritic knee [44]. A study of 214 cases with a mean follow-up of 38 months showed that no patients required revision of either component for loosening, wear, or instability, which helps support the efficiency of KA in TKA [45]. However, this study only analyzed a cohort of TKA aligned kinematically and did not compare with a control group.

There has been, and there still is a concern, that varus alignment of the tibial component might compromise clinical results and place implants at a higher risk for loosening [21, 46–48]. KA, which increases the varus angle of the tibial implant, could result in different complications. However, focusing on implant survivorship, in contrast to the original concerns, KA TKA may not lead to an increased early rate of loosening [12, 20, 49, 50] considering short and mid-term follow-up. One explanation is that KA TKA restores the JLOA of the native knee, which is parallel to the floor when standing, thereby mitigating overload of the medial and lateral tibial compartment [49, 51]. Moreover, Howell et al. reported no significant difference in complication rate for TKA with KA compared to TKA with MA even at long term follow-up [52]. Laende et al. [40] showed a similar tibial component migration in the KA group compared to the MA group, even with a tibial component placed in varus. However, precautions must be taken when placing component in a kinematical alignment. Nedopil et al. highlighted a strategy for lowering the risk of tibial component loosening when performing KA which is to set the tibial component parallel to the flexion-extension plane (slope) and varus-valgus plane of the native joint line [53].

In KA TKA, the aim was to implant components in a personal position re-creating the anatomy of the pre-arthritic articular surface of each patient chosen. KA allowing a close match with the native patient anatomy and the soft tissue envelope of the knee potentially, it will potentially improve ligament balancing and minimize the need for soft tissue releases and decreases the bone resection [38, 41, 54, 55]. Moreover, Blakeney et al. showed that the knee kinematics of patients with kinematically aligned TKAs more closely resembled that of normal healthy controls than that of patients with mechanically aligned TKAs [56]. Thus, a return to normal gait parameters after TKA will potentially lead to improved clinical outcomes with greater patient satisfaction. Consequently, KA may be considered as a safe procedure based on our review and the literature.

Several limitations should be discussed. Firstly, we only found five relevant studies that compared KA with MA in TKA with a minimum follow-up of 2 years. Secondly, despite a minimum follow-up of 2 years, the follow-up periods were generally too short to determine long-term longevity and survival. Thirdly, the operative method and instrumentation used to achieve kinematic alignment was not identical in all the studies, ranging from the use of patient-specific instruments with custom cutting guides to computer navigation or robotic assisted instrumentation. Furthermore, the target alignment in KA groups was not well defined for each study. However, radiological alignment results in KA groups appeared to be comparable for all studies. And lastly, the sample size in each group was relatively small.

## Conclusion

In conclusion, we found that KA in TKA achieved clinical and radiological results similar to those of MA. The complication rate was not increased for KA TKAs. The present review suggests that KA is an acceptable and alternative alignment to MA. Studies with longer follow-up and larger cohorts are required to prove any benefit of the KA technique over MA technique.

## Conflict of interest

Prof. Sébastien Lustig has performed consultancy work for Medacta, Heraeus, Corin, Amplitude, Groupe Lépine, Depuy Synthes, Smith & Nephew, Stryker. Prof. Sébastien Lustig and Prof. Elvire Servien receive institutional research support from Corin and Amplitude. Prof. Sébastien Lustig is a board member of KSSTA and Maitrise Orthopédique. The other authors declare that they have no conflicts of interest.
